# Role of Ureteroscopy (URS) and Stone Treatment in Patients with Recurrent UTIs: Outcomes over a 10-Year Period

**DOI:** 10.3390/jcm12103591

**Published:** 2023-05-22

**Authors:** Francesco Ripa, Virginia Massella, Andrea Ong, Mriganka Mani Sinha, Amelia Pietropaolo, Bhaskar K. Somani

**Affiliations:** Department of Urology, University Hospital Southampton NHS Foundation Trust, Southampton SO16 6YD, UK; francesco.ripa@uhs.nhs.uk (F.R.); vi_m@hotmail.com (V.M.); andreaong94@gmail.com (A.O.); mrigankamani@gmail.com (M.M.S.); ameliapietr@gmail.com (A.P.)

**Keywords:** urinary tract infection, recurrent UTI, ureteroscopy, kidney stones, urosepsis

## Abstract

*Background*. The study aimed to assess whether the eradication of kidney stones might result in a substantial reduction in the onset of recurrent UTIs. *Methods*. We selected all the patients who underwent ureteroscopy (URS) for stone disease between 2012 and 2021, with either a history of recurrent UTIs (rUTIs), urosepsis or pre-operative positive urine culture (UC). Data included patient demographics, microbiological data, stone parameters, stone-free and infection-free rates (SFR and IFR, respectively) at follow-up, defined as fragments <2 mm at imaging and the absence of symptoms and urine-culture-proven UTI. *Results*. Overall, 178 patients were selected. The median age was 62 years. The median cumulative stone size was 10 mm (7–17.25), and the commonest locations were the lower pole (18.9%) and proximal ureter (14.9%). The overall stone-free rate at follow-up was 89.3%. The IFR at 3 months was 88.3%. As follow-up duration increased, the IFR reduced to 85.4%, 74.2%, 68% and 65% at 6, 12, 18 and 24 months, respectively. Patients who had infection recurrence were more likely to present stone persistence or recurrence compared to those who were infection-free at follow-up (20% vs. 4.4%, *p* = 0.005). *Conclusions*. SFR after URS is a significant predicting variable for the likelihood of infection-free status at follow-up in patients with an rUTI or positive UC at the time of URS.

## 1. Introduction

Trends in the incidence and prevalence of kidney stone disease (KSD) suggest a global increase in this condition over the last few decades, despite regional differences [1]. Consequently, its healthcare-associated economic burden has been estimated to exceed USD 2 billion annually in the US [2] and between GBP 190 million and GBP 324 million in England [3].

Kidney stone disease (KSD) and urinary tract infections (UTI) are two commonly coexistent conditions. The causative link between these is yet under investigation [4,5]. On the one hand, patients presenting with KSD are at higher risk of developing a UTI compared to non-stone-formers [6]; the prevalence of UTI in patients with KSD might be as high as 36% [7], and rates of bacteremia are higher in patients with urolithiasis compared to those without [8]. On the other hand, UTI has an evident causative role in the pathogenesis of struvite (magnesium ammonium phosphate) stones, frequently combined with calcium phosphate and calcium oxalate stones [9]. The management of infection stones may be challenging in the clinical practice setting, especially due to the high stone burden, significant recurrent rates and the risk of long-term chronic kidney disease. The treatment of infection stones comprises both antibiotic therapy and surgical clearance, and it is associated with good stone-free rates (SFR), low recurrence rates and reduced morbidity and mortality [10]. Despite this, infection stones account only for 10–15% of overall stones. Emerging evidence highlighted the possible causative role of bacteria also in the formation of metabolic calcium-containing stones [11].

Patients with recurrent UTIs and the concomitant presence of KSD may benefit from a proactive treatment of stones, irrespective of stone composition, since it could reduce the risk of infection during the follow-up after the surgical treatment [12,13,14]. However, it is difficult to quantify the beneficial effect of stone treatment on the prevention of urinary tract infections since rates of infection-free status (IFR) vary among different studies.

In a previous study from our team [15], we hypothesized that the presence of stones or residual stone fragments might act as an infective focus in patients with recurrent UTIs or a positive urine culture prior to surgical intervention. Among 103 patients submitted to ureteroscopy, eighty-one patients (79%) had a positive pre-operative urine culture (UC), while 22 patients (21%) reported a history of recurrent UTIs. In that study, we followed patients up to 12 months after ureteroscopy and reported a decreasing trend in infection-free rates from 88% at 3 months to 71% at 12 months; additionally, we highlighted the relationship between stone recurrence and UTI recurrence in this group of patients.

The aim of the current study was to analyze and correlate the outcomes of ureteroscopy (URS) on stone clearance (SFR) and infection clearance (IFR) with a longer follow-up (24 months) in a larger cohort of patients with KSD presenting with recurrent UTIs or positive urine culture or urosepsis.

## 2. Materials and Methods

The study was registered in our hospital (audit 6901), and data were collected prospectively on consecutive patients with kidney stones and UTIs. A valid written informed consent was obtained from all the patients, and data collection was performed independently by members of the team not involved in the original surgical procedure. We further analyzed and cross-checked it using electronic health records, patient correspondence and discharge summary.

In a prospective database, all the patients submitted to semirigid or flexible ureteroscopy for stone treatment in our tertiary university hospital between 2012 and 2021 (10 years) who met at least one the following inclusion criteria were evaluated:

- Positive urine culture collected during pre-assessment investigations.
- History of recurrent urinary tract infections (rUTI), defined according to EAU Guidelines as recurrences of UTIs with a frequency of at least three UTIs per year or two UTIs in the last six months [16].
- Urosepsis as clinical presentation, defined according to The Third International Consensus Definitions for Sepsis and Septic Shock (Sepsis-3) [17] as a life-threatening organ dysfunction caused by a dysregulated host response to infection.
- Patients treated for the above condition with ureteroscopy and a minimum follow-up of 24 months.

Selected patients included the cohort of patients submitted to ureteroscopy between March 2012 and July 2016, analyzed in the previous study from our group [15] with a median follow-up of 12 months.

Patients’ demographic characteristics, pre-operative renal function (expressed as eGFR and calculated according to the CKD-EPI Creatinine Equation, 2021) and stone characteristics (size, location and composition) were collected from electronic personal health records. A standardized approach was used during semirigid and flexible ureteroscopy for stone treatment, as previously described [18].

Our approach relies on a standardized surgical approach for each procedure, including a semirigid ureteroscopy in all cases, laser lithotripsy of ureteric stones if present, and a subsequent flexible ureteroscopy to explore the renal cavities and treat the renal stones. We routinely perform the removal of small stone fragments through an endoscopic basket for stone composition analysis.

Most cases were performed as day-case procedures. A temporary ureteric stent was placed for 1–3 weeks if deemed useful and subsequently removed via a flexible cystoscopy in an outpatient setting. Intra- and post-operative variables were also described, such as the operative time, use of a ureteral access sheath (UAS), insertion of a JJ ureteric stent, length of hospital stay and post-operative complications according to Clavien-Dindo classification [19] (Table 1). In all cases, a Ho:YAG laser system (Lumenis, Inc. San Jose, CA, USA) was used, either with fragmentation or dusting settings, with a 272-μm laser fiber (Lumenis, Inc.). In the majority of cases, at least one stone fragment was collected by endoscopic basket extraction and sent for stone composition analysis. Whenever a pre-operative urine culture was available, the causative organism was assessed and adequately treated before surgery with antibiotics, according to local antimicrobial guidelines. SFR was evaluated as endoscopically stone-free at the end of the procedure, and fragments <2 mm at 2–3 months imaging follow-up [20] with either XR KUB or Urinary Tract USS for radiological opaque or lucent stone, respectively, and with CT KUB for any diagnostic or decision dilemma. Infection-free rate (IFR) was evaluated at follow-up as an absence of symptoms and urine-culture-proven urinary tract infection. Positive urine culture was defined as bacterial or yeast growth ≥10^5^ CFU in a urine sample. The time to UTI recurrence from the time of surgery was also assessed. Follow-up intervals were set a priori at 3, 6, 12, 18 and 24 months from surgery.

We scheduled fixed-term follow-up appointments at 3, 6, 12, 18 and 24 months for patients submitted to ureteroscopy for stone removal to check on symptoms (pain, infections, haematuria) after surgery and to radiologically assess their stone-free status (with either XR KUB or USS KUB in case of radiopaque or radiolucent stones, respectively). If a patient was complaining of pain or UTI symptoms during follow-up, we performed a urine culture to confirm or exclude the presence of infection and treat the UTI if necessary. Due to the wide clinical variability, the pros and cons of conservative management vs. re-intervention were discussed with each patient according to the persistence of residual stone fragments and/or the persistence of infective symptoms.

SFR and IFR were correlated during the follow-up. Statistical analysis was performed using SPSS version 27.0.

## 3. Results

A total of 178 patients submitted to URS for stone treatment met the inclusion criteria (Table 1). All the procedures were performed between 2012 and 2021. The male-to-female ratio was 1:1.2, and the median age was 62 years (IQR 47–76). Pre-operative and post-operative mean eGFR were 62.6 and 60.1 mL/min, respectively. Among these patients, 106 (59.5%) presented with urosepsis, while 72 (40.5%) had rUTIs. A pre-operative positive urine culture was present in 29.8% of cases, and among the causative organisms *E. coli* was the most prevalent pathogen (54.1%), followed by yeast infections (16.2%).

The median single and cumulative stone size was 9 mm (IQR 6–12) and 10 mm (IQR 7–17.3), respectively. Eight percent of patients presented with bilateral stones. The most common primary stone locations were the lower pole (18.9%), proximal ureter (14.9%) and pelvic-ureteric junction (13.5%). The primary stone composition analysis revealed a prevalence of calcium oxalate mono/dihydrate stones (57.7%) and calcium phosphate carbonate stones (26.9%); the most common secondary stone composition was calcium phosphate carbonate (57.7%) and struvite (25.4%) stones. Eighty-four patients had a pre-operative stent (47.2%).

An intra-operative variables analysis showed a median operative time (OT) of 38 min (IQR 22–57.5); a ureteral access sheath was placed in 37.7% of cases; the rate of post-operative ureteric JJ stent insertion was 78.1%. The median indwelling time of ureteric JJ stent was 13 days (IQR 4.25–22). The mean length of hospital stay was 0.96 days (IQR 0–1).

Twelve post-operative complications (6.7%) were reported and described according to Clavien-Dindo classification: one grade I (acute urinary retention); eight grade II (four cases of UTIs and four cases of post-operative urosepsis requiring IV antibiotics); one grade III (displacement of JJ ureteric stent requiring replacement); two grade IV complications (urosepsis requiring ICU admissions).

We observed ten infective complications in the post-operative period (four cases of UTIs and six cases of urosepsis). These cases were considered post-operative events, and the overall rate of early post-operative recurrences (positive urine culture within the first 30 days) was 6.2% (11 cases). However, we did not include infective post-operative complications (10) in the cohort of UTI recurrence at 3 months.

The outcomes of URS on stone-free rates and infection-free rates are shown in Table 2. Table 2 shows the number and percentage of patients who were stone-free at follow-up or had a stone recurrence at 3, 6, 12, 18 and 24 months, and the number and percentage of patients who were infection-free or had a UTI recurrence at 3, 6, 12, 18 and 24 months, respectively.

No patient was lost at follow-up, and the median follow-up time was 25 months (IQR 6–57). At 3 months, SFR was 92.1%, while not-stone-free patients were 7.9%. Among patients who were stone-free at 3 months, we reported the relative proportion of patients who experienced stone recurrence during follow-up (1.2%, 3%, 6.7% and 12.2% at 6, 12, 18 and 24 months, respectively).

IFR at 3 months was 88.8% (*p* = 0.06). At 6 months, SFR was 91%, and IFR was 85.4% (*p* < 0.001). At 12 months, SFR was 89.3%, while IFR was 74.2% (*p* < 0.001). At 18 months, SFR was 86%, and IFR was 68% (*p* < 0.001). Finally, at the 24-month follow-up, SFR was 80.9%, and IFR was 65.2% (*p* < 0.001).

We analyzed the effect of additional risk factors on the outcome of the infection-free rate after ureteroscopy. There was a statistically significant difference in the mean age between the infection-free group and the group of patients who were not infection-free. Patients who were not free from UTI at FU were 8.6 years older (95% CI 0.02–17.1) compared to those who were cleared from infections after ureteroscopy (*p* = 0.04). This might confirm the role of a younger age in achieving better infection-free rates after surgery compared to older patients with recurrent urinary tract infections and the concomitant presence of stones. The presence of diabetes was not associated with a higher proportion of UTI recurrence (*p* = 0.37). Finally, we did not report any significant difference in the relative proportion of different primary or secondary stone compositions among patients who were UTI-free at follow-up and those who had UTI recurrence (*p* = 0.7).

Table 3 highlights the relative proportion of stone-free rates and infection-free rates at follow-up. Among patients that were not stone-free after URS, IFR at 3 months of follow-up was markedly lower (63.6%) compared to those who were cleared of stones (90.4%, *p* < 0.006). Additionally, among patients who had infection recurrence at 3 months, 20% of them also had a stone persistence/recurrence, vs. 4.4% (*p* = 0.005) among those who were infection-free. The median time to UTI recurrence after URS was 8.1 months (IQR 2–17), showing early onset of infection in those who recurred.

There was no significant difference in the relative proportion of infection-free rates between patients presenting with recurrent UTIs and patients presenting with urosepsis as the first clinical presentation. Due to the heterogeneity of the inclusion criteria, we wanted to assess separately the outcomes of ureteroscopy on stone-free rates and infection-free rates in the different patient groups (sepsis at presentation, recurrent UTIs and positive pre-operative urine culture groups). The infection-free rate at 3 months was similar among patients presenting with urosepsis and recurrent urinary tract infections (88.3% vs. 85.7%, respectively, *p* = 0.6). Similarly, infection-free rates were not significantly different between patients with a positive pre-operative urine culture and those with clear urine (84.9% vs. 90.4%, respectively, *p* = 0.3).

We observed similar SFR between patients with temporary post-URS ureteric stents and those without (94.2% vs. 91.2%, respectively, *p* = 0.5). The rate of complications was comparable between patients with or without a post-operative stent (8.3% vs. 5.9%, respectively, *p* = 0.6).

## 4. Discussion

### 4.1. Meaning of the Study

The primary aim of this study was to analyze whether a correlation existed between stone clearance after ureteroscopy for KSD and patients’ infection-free status, defined as the absence of symptoms and a negative urine culture during follow-up. Our intention was to assess whether the persistence of stones might act as a risk and promoting factor for the recurrence of UTIs after ureteroscopy and to evaluate the time frame between the surgical intervention and the first recurrence of urinary infection. Based on the available literature evidence, we hypothesized that the active treatment and removal of kidney stones could lead to the resolution—at least temporary—of UTI episodes in patients presenting with either recurrent UTIs or urosepsis.

We believe our findings could suggest that stone-free status after ureteroscopy is a significant predictive variable for infection-free status at follow-up in patients with either recurrent UTI episodes or urosepsis associated with concomitant kidney stones, and stone clearance could result in the persistence of infection-free status in the majority of patients.

### 4.2. Comparison with Other Studies

Due to technological improvements, the role of ureteroscopy in stone treatment has expanded over the last decades, proving its safety and efficacy even in difficult clinical scenarios and for large renal stones [15,21]. Few studies have focused on the outcomes of URS for renal stones in patients with recurrent UTIs or positive urine culture in order to investigate the role of an active stone treatment in reducing the risk of urinary infections.

A retrospective study from the US reported 48% of infection-free status after stone treatment vs. 52% of UTI recurrence in a group of 120 patients with rUTIs and a nonobstructive renal stone, with a mean follow-up of 14 ± 3 months [12]. After multivariate analysis, black ethnicity, hypertension and *E. coli* infections were associated with unsuccessful clearance of recurrent UTIs after surgery. The authors concluded that an attempt at stone clearance should be offered to patients with recurrent UTIs.

Schembri et al. [22] examined the outcomes of ureteroscopy on symptom resolution in 109 patients presenting with a single symptomatic stone <10 mm in any location. According to the symptoms, patients were subdivided into those with pain, urinary tract infection (UTI) and hematuria. Among 28 patients presenting with a culture-proven UTI, a complete resolution was observed in 85.7% of the cohort. Additionally, no significant differences in symptom resolution between patients with stones <7 mm and those with stones of 8–10 mm were found; the cessation of symptoms was not associated with a specific stone composition. Based on this evidence, the authors recommended that patients with symptomatic, small renal stones should be offered endoscopic treatment.

An 89% rate of infection clearance after stone extraction was reported in a retrospective study including 46 patients with recurrent UTIs and a non-obstructive, non-infectious renal stone [14], with a median post-operative follow-up of 2.9 years. SFR for ureteroscopy was 63% vs. 65% for PCNL, with or without ureteroscopy. The presence of residual stones was a significant risk factor for infection recurrence.

Finally, a recent systematic review on infection-related post-ureteroscopy complications [23] highlighted the presence of a positive pre-operative UC or a prior history of UTIs as predisposing factors for urosepsis following ureteroscopy. While endourology and ureteroscopy are relatively safe, mortality has been reported, which is specifically related to post-procedural sepsis [24,25].

The relationship between renal stones and urinary tract infections has been widely demonstrated. Kidney stone formers are known to be exposed to a higher risk of urinary tract infections during follow-up. Despite all the recent evidence, the etiopathogenetic role of bacteria in stone aggregation and growth is yet to be clarified, and it extends beyond the causative role of urea-splitting bacteria in the onset of infective struvite stones, also involving the most common calcium-containing stones. Active treatment of stones in patients with recurrent UTIs might be beneficial and prolong infection-free periods.

### 4.3. Strengths, Limitations and Areas of Future Research

The strength of our study lies in the large cohort of patients who underwent a single-type and standardized procedure (ureteroscopy) performed by a single surgeon (BS) with a structured pre-assessment pathway and a preset follow-up at regular intervals. Data were collected in a prospective manner, and the follow-up after surgery was at 24 months.

Despite this, our study is not devoid of limitations. Primarily, the patient cohort was not homogeneous since it comprehended two clinically different scenarios (recurrent UTIs and urosepsis as the first presentation). Secondly, there was no uniformity in the assessment of post-operative stone-free status because patients were offered either XR KUB or urinary tract USS during follow-up. Finally, we did not perform an extensive comparison and multivariable analysis of clinical data between patients who recurred and patients who were free from infections.

Discordant data regarding infection-free rates after surgery point out that the relationship between kidney stones and recurrent UTIs is complex, multifaceted and yet to be fully understood. Several factors are involved, including both a patient’s and pathogen’s characteristics, local antibiotic treatment guidelines, surgical expertise, metabolic assessment and follow-up protocol. However, our results seem to be in line with emerging evidence on the beneficial effect of active stone treatment in patients with recurrent UTIs. Further larger prospective and multi-center studies with standardized inclusion criteria and assessment methods are recommended.

## 5. Conclusions

Patients with recurrent UTIs or urosepsis as clinical presentation who are stone-free after ureteroscopy demonstrate higher infection-free rates during follow-up compared to those with residual stone fragments. This beneficial effect is evident after surgery and is not correlated to the stone composition analysis.

## Figures and Tables

**Table 1:** Descriptive characteristics of the population. eGFR—estimated glomerular filtration rate; PUJ—pelvic ureteric junction; OT—operative time; UAS—ureteral access sheath; LOS—length of hospital stay; IQR—interquartile range; SD—standard deviation; UTI—urinary tract infection; Cumulative stone length refers to the sum of the largest diameters of all the stones.

Patient Demographic	
Males (N, %): Females (N, %)	80 (44.8%); 98 (55.2%)
Age (median, IQR)	62 (47–76)
BMI (median, IQR)	26.6 (23.1–31.8)
Sepsis/pyelonephritis (N, %)	106 (59.5%)
Recurrent UTI (N, %)	72 (40.5%)
Positive pre-operative urine culture (N, %)	53 (29.8%)
Pre-operative eGFR, ml/min (mean, SD)	62.57 (20.1)
Post-operative eGFR, ml/min (mean, SD)	60.09 (22.7)
**Stone characteristics**	
Largest diameter, mm (median, IQR)	9 (6–12)
Cumulative stone length, mm (median, IQR)	10 (7–17.25)
**Side**	
Left side (N, %): Right side (N, %)	72 (40.2%): 92 (51.8%)
Bilateral (N, %)	14 (8%)
**Primary Location**	
Distal ureter (%)	14.8
Mid ureter (%)	10.8
Proximal ureter (%)	14.9
PUJ (%)	13.5
Lower pole (%)	18.9
Mid pole (%)	6.8
Upper pole (%)	7.4
Multiple stones	12.9
**Operative data**	
Median OT (min, IQR)	38 (22–57.5)
UAS placement (%)	37.70%
Post-operative JJ stent insertion (%)	78.10%
Median JJ stent indwelling time (days, IQR)	13 (4.25–22)
Mean LOS (days, IQR)	0.96 (0–1)
**Post-operative complications**	
Overall (*n*, %)	12 (6.7%)
Clavien I	1
Clavien II	8
Clavien III	1
Clavien IV	2

**Table 2:** Outcomes of ureteroscopy on stone clearance and infection clearance at 3, 6, 12, 18 and 24 months of follow-up. UTI—urinary tract infections.

N (%) of Patients					
*n* = 178	3 Months	6 Months	12 Months	18 Months	24 Months
Stone-free	164 (92.1%)	162 (91%)	159 (89.3%)	153 (86%)	144 (80.9%)
Not stone-free	14 (7.9%)	16 (9%)	19 (10.7%)	25 (14%)	34 (19.1%)
(Stone recurrence)	-	(2, 1.2%)	(5, 3%)	(11, 6.7%)	(20, 12.2%)
Infection-free	158 (88.8%)	152 (85.4%)	132 (74.2%)	121 (68%)	116 (65.2%)
UTI recurrence	20 (11.2%)	26 (14.6%)	46 (25.8%)	57 (32%)	62 (34.8%)
Concurrent Stone- and UTI-free	*p* = 0.06	*p* < 0.001	*p* < 0.001	*p* < 0.001	*p* < 0.001

**Table 3:** Relative proportion of stone-free rates and infection-free rates at 3 months.

		Stone-Free at 3 Months			
		No	Yes	Total	
Infection-free at 3 months	No	4 (20%)	16 (80%)	20	*p* = 0.006
	Yes	7 (4.4%)	151 (95.6%)	158	
		Infection-free at 3 months			
		No	Yes	Total	
Stone-free	No	4 (36.4%)	7 (63.6%)	11	*p* = 0.006
	Yes	16 (9.6%)	151 (90.4%)	167	

## Data Availability

The data presented in this study are available (as anonymized data) on reasonable request from the corresponding author. The data are not publicly available due to having patient specific data.

## Abbreviations

eGFR—estimated glomerular filtration rate; PUJ—pelvic ureteric junction; OT—operative time; UAS—ureteral access sheath; LOS—length of hospital stay; UTI—urinary tract infection; URS—ureteroscopy; SFR—stone-free rate; IFR—infection-free rate; KSD—kidney stone disease; XR KUB—XR kidneys ureters bladder; CTKUB—CT kidneys ureters bladder; IQR—interquartile range; ICU—intensive care unit; PCNL—percutaneous nephrolithotomy.
